# Dual-resonance sensing for environmental refractive index based on quasi-BIC states in all-dielectric metasurface

**DOI:** 10.1515/nanoph-2022-0776

**Published:** 2023-02-21

**Authors:** Wenjie Chen, Ming Li, Wenhao Zhang, Yuhang Chen

**Affiliations:** Department of Precision Machinery and Precision Instrumentation, University of Science and Technology of China, Hefei 230027, China; Key Laboratory of Precision Scientific Instrumentation of Anhui Higher Education Institutes, University of Science and Technology of China, Hefei 230027, China

**Keywords:** BIC, dielectric metasurface, refractive index, resonance, sensing stability

## Abstract

Metasurface provides a novel way to modulate light energy at specific wavelengths, namely resonances, where there is a sharp drop in the transmission spectrum. Based on the relationship between the resonant position and the environmental condition, various refractive index detection methods have been developed. However, the resonance spectrum is strongly affected by the environmental and instrumental fluctuations, and current researches usually focus on the improvement of a single sensing performance metric, such as the Q factor, sensitivity, detection range, etc. In this work, we proposed an all-dielectric metasurface for environmental refractive index sensing based on quasi-BIC with an enhanced stability, simultaneously taken into account an enlarged detection range, a high Q factor and a relatively high sensitivity. With this designed metasurface, dual-resonance sensing is realized because the interval between the two resonance peaks in the transmission spectrum decreases near linearly with the environmental refractive index. We experimentally demonstrated that compared to traditional single-resonance sensing, the errors caused by environmental and instrumental fluctuations can be minimized, and the stability can be improved. This metasurface has great potential for applications such as refractive index sensing, concentration detection, biomacromolecule identification, and cancerous cell screening.

## 1 Introduction

Refractive index sensing is in high demand in industrial production, biochemical analysis, food safety inspection, clinical diagnosis, and environmental detection. Various environmental refractive index detection methods have been developed based on the relationship between the resonant position and the environmental refractive index. Optical fiber sensors have been at the forefront of this research field because of their low loss, high sensitivity, and anti-electromagnetic interference ability. Among these, long-period fiber gratings (LPFG) [1, 2], Bragg fiber gratings (BFG) [3] and interferometric optical fibers [4–8] are the most commonly used. Specifically, the fiber core of LPFGs has a periodic refractive index, which can respond to environmental changes such as tension, temperature and refractive index. Interferometric optical fibers are primarily based on Mach–Zehnder, Michelson, Sagnac, and Fabry–Perot interference. However, aside from the long fiber length and fabrication difficulty, optical fiber sensors are limited because of their relatively low stability, and the detection range for refractive index is also insufficient, typically limited to 0.0001–0.1 [2, 3, 8], [9], [10], [11], [12].

High quality (Q) factor, which describes the capacity for storing energy and is also measured by figure of merit (FOM) in some cases, is essential for the sensing system. The development of metamaterials has provided new prospects for sensing with high sensitivity as well as high Q factor. Artificial microstructures can capture electromagnetic waves at specific frequencies, providing a platform for enhancing the light–matter interaction. Metal nanoparticles can generate local surface plasmon resonances (LSPRs), and thus produce a significant near-field enhancement at the nanoscale. LSPRs are environmentally sensitive and are widely applied in surface-enhanced Raman spectroscopy, plasma lasers, nanoantennas, biosensors, and environmental sensors [13–19]. Recently, there also emerges mind-controlled metasurfaces via brainwaves formed by metal layers [20]. However, metal-based metasurfaces suffer from low efficiency, and their corresponding Q factors are limited due to the intrinsic Ohmic dissipation of metals. In contrast, dielectric metasurfaces can overcome this limitation [21–24]. Representative examples of achieving optical resonances with high Q-factors are realized by multiple reflections and multiple forming layers. Existing studies on multiple-reflections fall into various microcavities, such as Fabry–Perot, photonic crystals, and whispering gallery mode (WGM) cavities [25–28]; however, they are limited by cumbersome integrated systems, fabrication and alignment difficulties, diffraction loss, and susceptibility to external conditions. Moreover, a reasonable combination of different materials is conducive to fully exploiting the advantages of each material, providing a narrower transmission dip, higher sensitivity, and chemical discrimination ability [29–32]. However, the fabrication process is complicated, and the radiation losses are still unavoidable. Over the past several years, enhanced resonances for refractive index detection also have sprung up based on mode coupling [33, 34]. Despite their high Q factor as well as the high sensitivity, it is usually difficult to adjust the resonance wavelength and the detection is thus extremely limited.

Recently, researchers have proposed the concept of bound states in the continuum (BIC) to achieve exceptionally sharp spectral resonance. Unlike traditional bound states, BIC is completely decoupled from the radiative continuum though located in it [35]. Theoretically, BIC has an infinite Q factor; however, it cannot be excited directly by the incident wave owing to its decoupling from the radiative wave. In addition, BIC only exists in ideal lossless structures with zero permittivity, and is not possible to be detected because of its zero spectral linewidth. In practice, BIC is transformed into quasi-BIC with extremely high but finite Q factors [36–44], which can be accessed usually by breaking the in-plane inversion structural symmetry [35, 38], for example, using nanocubes with a missing angle [37], nanodisks with asymmetric holes [43], split-ring structures [44], and pairs of inversely tilted elliptic pillars. Quasi-BIC has been employed in nonlinear harmonic generation [40], biosensing [41], and topological optics.

We summarized a few examples and the corresponding characterizations of refractive index sensing adopted in previous works as listed in Table 1. However, influenced by the fluctuation of the temperature, humidity, and intrinsic drift of the instrument, the obtained spectrum will exhibit an artificial shift, and traditional single-resonance sensing may therefore be inaccurate and unreliable. This problem can be solved by converting single-resonance sensing into dual-resonance sensing, and tracing the resonant position is transformed into analyzing the spacing of the two resonance peaks accordingly. When the external conditions fluctuate, the two resonance peaks simultaneously exhibit a red/blue shift; thus, the spectral shift caused by the environment and instrument fluctuation is reduced to some extent. The dependence of the resonance wavelengths on the environmental refractive index can be explained by first-order perturbation theory [45]. However, there are few reports on the detection of the environmental refractive index using multiple-resonance metasurfaces by monitoring the changes of the intervals between the resonance wavelengths. At the same time, previous works usually focus on the improvement of a single detection performance metric, which is difficult to simultaneously achieve a high Q factor, a large detection range and a high sensitivity.

**Table 1:** Detecting characterizations of different refractive index sensing methods.

Method	Sensitivity/nm·RIU^−1^	Q factor/FOM	Detection range	Reference
BFG	204	–	1.00018–1.00028	[3]
Mach–Zehnder interferometer	95,832	–	1.32–1.44	[7]
LSPR	1010	FOM = 108	1.333–1.417	[18]
Dielectric metasurface	161	–	1.32–1.38	[24]
Material combination	2929.1	FOM = 33.45	1.3334–1.3605	[32]
Mode coupling	–	Q factor = 350	1.333–1.415	[33]
This work	122.2	Q factor = 415	1.0–1.5	

**Figure 1:**
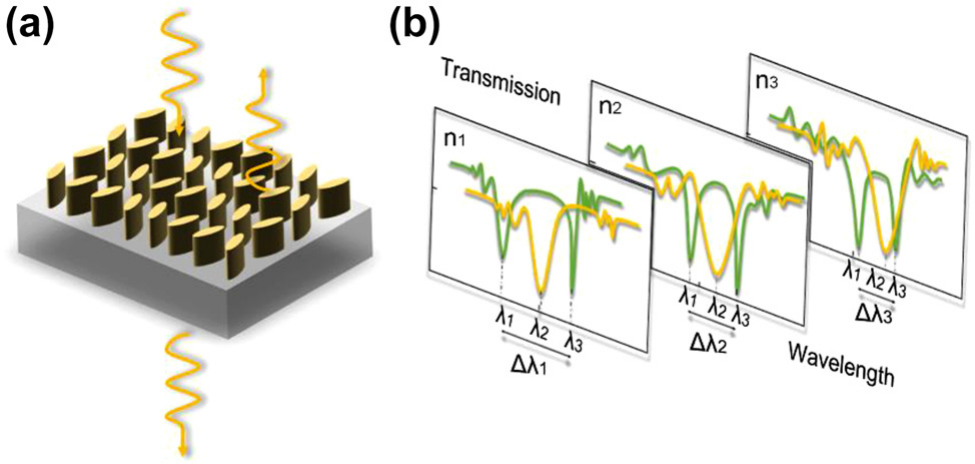
Schematic diagram of the metasurface for dual-resonance sensing. (a) Sketch of the metasurface. (b) Schematic diagram of the principle for single-resonance sensing (yellow curve) and dual-resonance sensing (green curve).

In this study, we present a metasurface for environmental refractive index sensing based on quasi-BIC, and experimentally demonstrate that through proper optimization of the height and the arrangement of the nanopillars, conventional single resonance sensing can be converted into dual-resonance sensing with a high Q factor, an expanded testing range, and a relatively high sensitivity simultaneously. In addition, the errors caused by environmental fluctuations can be significantly reduced. Figure 1(a) shows a sketch of the proposed metasurface, and Figure 1(b) depicts the general principle of single-resonance sensing (yellow curve) and dual-resonance sensing (green curve).

## 2 Metasurface design and optimization

### 2.1 Dual-resonance achieved by tuning nanopillar height

**Figure 2:**
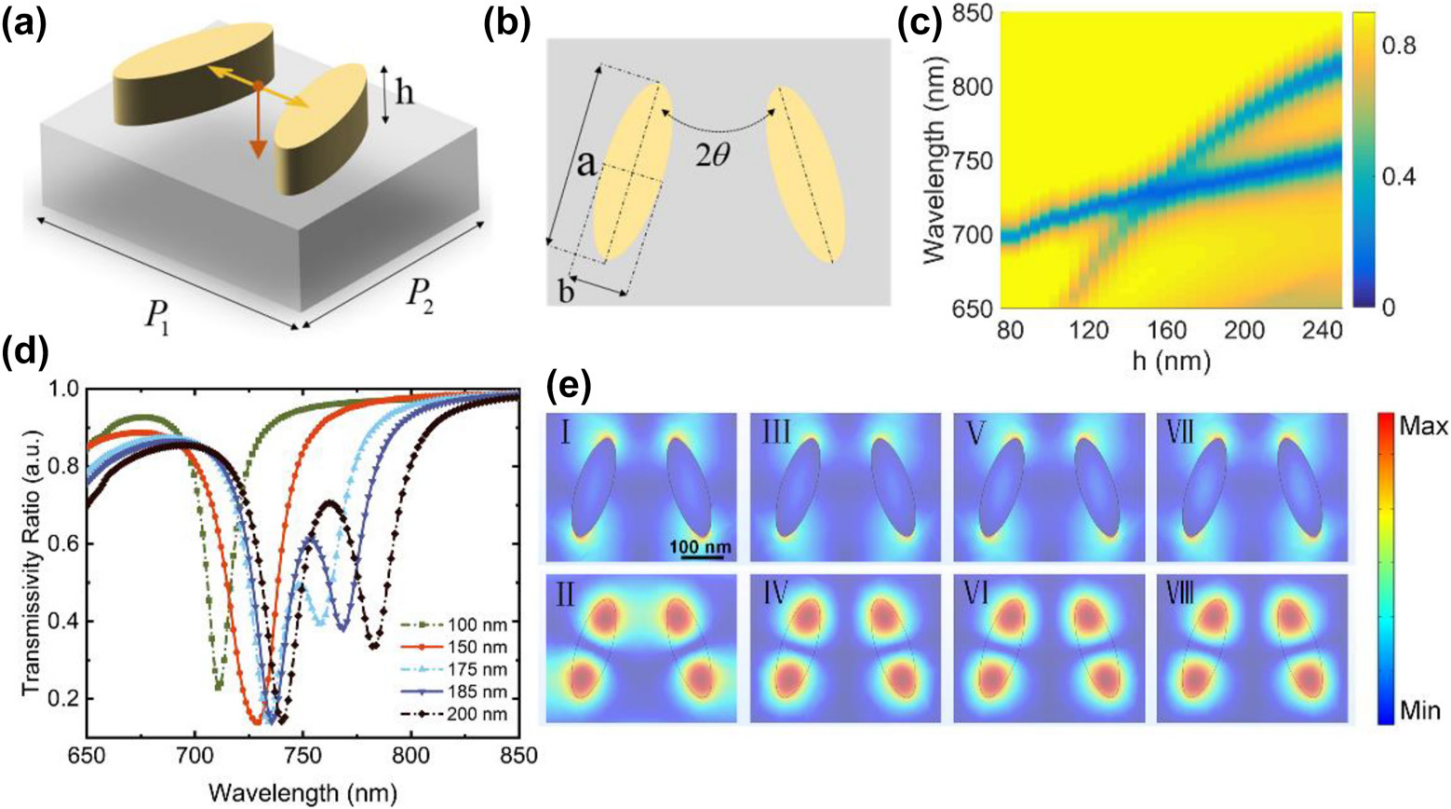
Transmission spectra and electric fields of the initial metasurfaces containing two nanopillars in one period. (a) And (b) schematic diagrams of the initial arrangements of the elliptical nanopillars. Specifically, *a* = 240 nm, *b* = 80 nm, *θ* = 17.5°, *P*_1_ = 450 nm, and *P*_2_ = 350 nm. (c) Dependence of the transmission spectra on the height of the nanopillars. (d) Transmission spectra of five metasurfaces containing nanopillars of 100 nm, 150 nm, 175 nm, 185 nm, and 200 nm height, respectively. (e) The corresponding electric fields of the eigenmodes in accordance with (d).

Owing to its high refractive index, low loss, and high regulatory capacity in the visible and near-infrared ranges, polycrystalline silicon (poly-Si) is widely used in the fabrication of metasurfaces. Our designed metasurface is composed of elliptic poly-Si nanopillars because nanopillars in this shape can increase the degree of freedom and improve the detection sensitivity with a relatively high anti-interference ability [46]. Considering the operating waveband of the experimental optical spectrometer (AvaSpec-2048-4, Avantes, Netherlands), the working wavelength band of the metasurface is designed to be approximately 700–900 nm. The refractive index for detection is set between 1.0 and 1.5, which covers that of common reagents such as glycerol, ethyl acetate, and benzyl alcohol. Initially, the designed metasurface contains two nanopillars in one period, as illustrated in Figure 2(a) and (b). Here, *h* is the height of the nanopillars, *a* and *b*, respectively, denote the major and minor axes, *θ* indicates the rotation angle between the major axis of the nanopillar and the *Y*-axis, and *P*_1_ and *P*_2_ are the periods along the *X*- and *Y*-axes. To satisfy the refractive index sensing with a high sensitivity and an enhanced Q factor, these parameters are optimized via parametric scanning, as detailed in Figure S1, Supplementary Material. Certainly, based on the same principle, we can extend the resonance peak positions to locate in mid-infrared or far-infrared wavebands by adjusting the sizes of the nanopillars, and the detection range for the refractive index can be tuned with nanopillars with varying sizes. Numerical simulations were carried out via finite-difference time-domain (FDTD) method where an incident light is polarized along the *X*-axis, and the refractive index of poly-Si was measured using an ellipsometer to reduce the simulation error caused by the deviations of material properties.

As depicted in Figure 2(c), the nanopillar height *h* is scanned from 75 nm to 250 nm, and the single resonance splits as *h* increases. The two resonant modes overlap at around *h* = 150 nm. To visualize this transition, we calculated the transmission spectra of five metasurfaces with different nanopillar heights, namely, *h* = 100 nm, 150 nm, 175 nm, 185 nm, and 200 nm, respectively. As illustrated in Figure 2(d), there is only one resonant mode for *h* = 100 nm and 150 nm, whereas two transmission dips appear in the spectra for the rest three metasurfaces. At the same time, the regulation depth increases as h increases, together with a red shift of the resonance wavelengths.

To further understand the mechanism of the single-resonance-splitting process, we calculated the electrical field intensity distributions of the corresponding eigenmodes, in the XY plane at the middle of the nanopillars of these five metasurfaces. As shown in Figure 2(e), panels I and II denote for the electric fields of the single eigenmode for metasurfaces of 100 nm and 150 nm height. Panels III and IV, V and VI, and VII and VIII are the electric fields of the two eigenmodes for metasurfaces of 175 nm, 185 nm and 200 nm height, respectively. From the electric fields, it can be found that the two resonances are driven by different eigenmodes, and the second eigenmode emerges as *h* increases. At the first eigenmode, more energy is concentrated outside of the two ends of the nanopillars. However, the energy is shifted to the inside of the nanopillars at the second eigenmode. The concentration of energy at the resonances contributes to the two sharp transmission dips in the transmission spectrum. Although the resonance wavelengths shift with different *h*, the electric intensity distributions of the eigenmodes do not change significantly. Specifically, the electric field is distorted when *h* = 150 nm and appears like the superposition of those of the two eigenmodes, which is the result of mode coupling at this position.

### 2.2 Optimization of nanopillar arrangement

**Figure 3:**
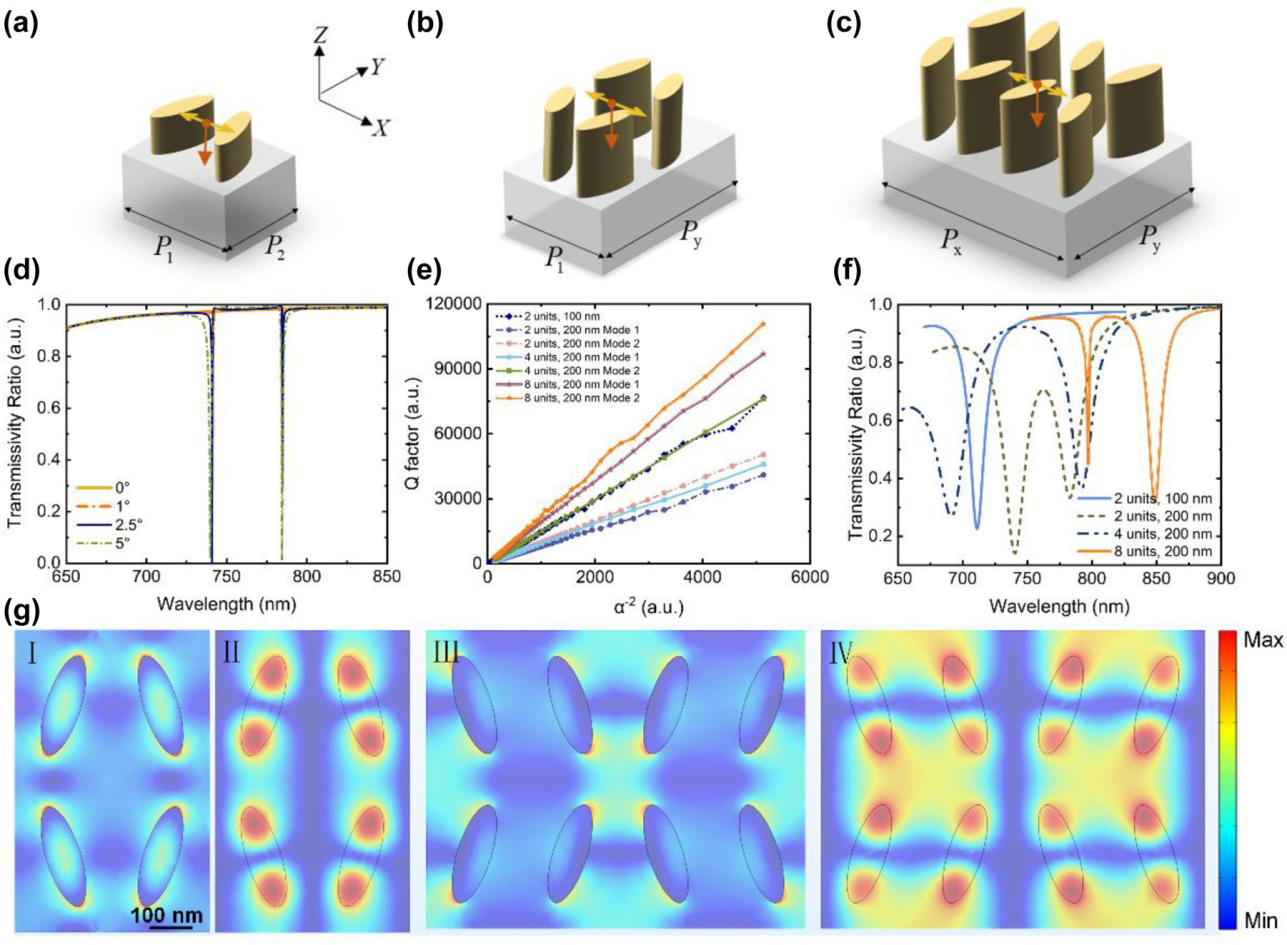
The corresponding transmission spectra and electric fields of metasurfaces with different nanopillar arrangements. (a)–(c) Illustrations of three arrangements of nanopillars, with 2, 4 and 8 elliptical nanopillar units arranged in one period. *P*_*x*_ and *P*_*y*_ are twice of *P*_1_ and *P*_2_, respectively. (d) The transmission spectra of metasurfaces composed of two 200-nm-height nanopillars in one period, with *θ* of 0°, 1°, 2.5°, and 5°, respectively. (e) The dependence of the Q factor on *α* in four metasurfaces. The nanopillars are considered ideally lossless in (d) and (e), and *α* is the sine of *θ*. (f) Transmission spectra of the four metasurfaces when considering the material loss. (g) The electric field maps of the two eigenmodes for metasurfaces containing four nanopillars of 200 nm height (I and II) and eight nanopillars of 200 nm height (III and IV) in one period, respectively.

Take into account the resonance wavelengths, the interval between the resonances, the regulation depth (reflected in the spectral peak contrast) and the fabrication availability, *h* is set as 200 nm to obtain the dual-resonance spectrum. Based on the metasurface containing two nanopillars each period, we then further change the way the initial nanopillars arranged in one period by mirroring or rotating each nanopillar cell to form a supercell with four or eight nanopillars to explore the corresponding sensing characterizations. Figure 3(a)–(c) depict the schematics of the nanopillar arrangement, in accordance with two, four and eight elliptical nanopillars evenly distributed with different orientations over one period. For metasurfaces containing four and eight nanopillars each period, the height of the nanopillars is 200 nm with the periods doubled accordingly, namely, *P*_*x*_ = 2*P*_1_ and *P*_*y*_ = 2*P*_2_. Other parameters are kept the same as the former.

Here, we set up four groups for comparison, i.e., the initial single-resonance group with two nanopillar units of 100 nm height each period, the group with two nanopillars of 200 nm height each period, and the two newly arranged groups with four 200-nm-height and eight 200-nm-height nanopillar units each period. According to the BIC concept, the bound states are restricted in a symmetry-protected system. However, energy will leak out when the in-plane symmetry is broken, namely when the rotation angle *θ* is nonzero. As an example, Figure 3(d) shows the transmission spectra of metasurfaces composed of two 200-nm-height nanopillars in one period, with *θ* of 0°, 1°, 2.5° and 5°, respectively. Clearly, when the symmetry is protected with *θ* = 0°, the transmission spectrum is flat with a high transmission ratio over the waveband. However, two sharp absorption peaks show up when the in-plane symmetry is broken. Moreover, as shown in Figure 3(e), the corresponding Q factors of the seven eigenmodes out of the four groups all decay quadratically with the asymmetry degree *α*, which is defined as the sine of *θ*. This characteristic further indicates that the resonances are driven by quasi-BIC, regardless of how the nanopillars are arranged [35, 47]. Compared to the three other groups with the same rotation angle *θ*, the Q factors of the two eigenmodes are enhanced greatly for the metasurface containing eight 200-nm-height nanopillars each period. The nanopillars are considered ideally lossless in Figure 3(d) and (e), and the Q factor is defined as the ratio of the real part to the imaginary part of the eigenfrequency in FDTD computations.

The Q factors can be extremely high when *θ* has a relatively low value with an ultra-precise fabrication process. However, an ultra-narrow transmission peak is usually hard to be detected with a resolution-limited spectrometer. Metasurface with a large *θ* also presents a higher tolerance to fabrication errors, and the corresponding transmission spectrum exhibits a larger modulation depth. In order to ensure that the sample preparation is feasible while obtaining a moderate modulation depth, *θ* is chosen as 17.5°. Figure 3(f) illustrates the transmission spectra of the four metasurfaces in air, and in correspondence with Figure 3(e), the metasurface containing eight nanopillars of 200 nm in one period presents a much sharper transmission dip at the first resonance, which leads to a higher Q factor. The electric fields of the corresponding two eigenmodes for metasurfaces containing four nanopillars of 200 nm height (I and II) and eight nanopillars of 200 nm height (III and IV) in one period are shown in Figure 3(g). Compared to those for metasurface containing two 200-nm-height nanopillars each period, the electric fields are distorted to some extent. The coupling between the adjacent nanopillars is increased, especially for the metasurface containing eight nanopillars of 200 nm height each period. The light energy is distributed unevenly and is more concentrated in one end of the nanopillars. The enhanced near-field coupling and the concentrated energy are responsible for the narrowed resonance peaks.

### 2.3 Simulated sensing performance

By adjusting the height of the nanopillars, a dual-resonance spectrum is obtained. Since the metasurface containing eight 200-nm-height nanopillar units in one period presents a sharper transmission dip, we adopt this metasurface as the target group for refractive index sensing. To illustrate that the dual-resonance detection driven by symmetry-broken nanopillars has enabled the metasurface with an improved Q factor along with a relatively high sensitivity, the single-resonance metasurface containing two 100-nm-height nanopillar units in one period is set as a control group for comparison.

**Figure 4:**
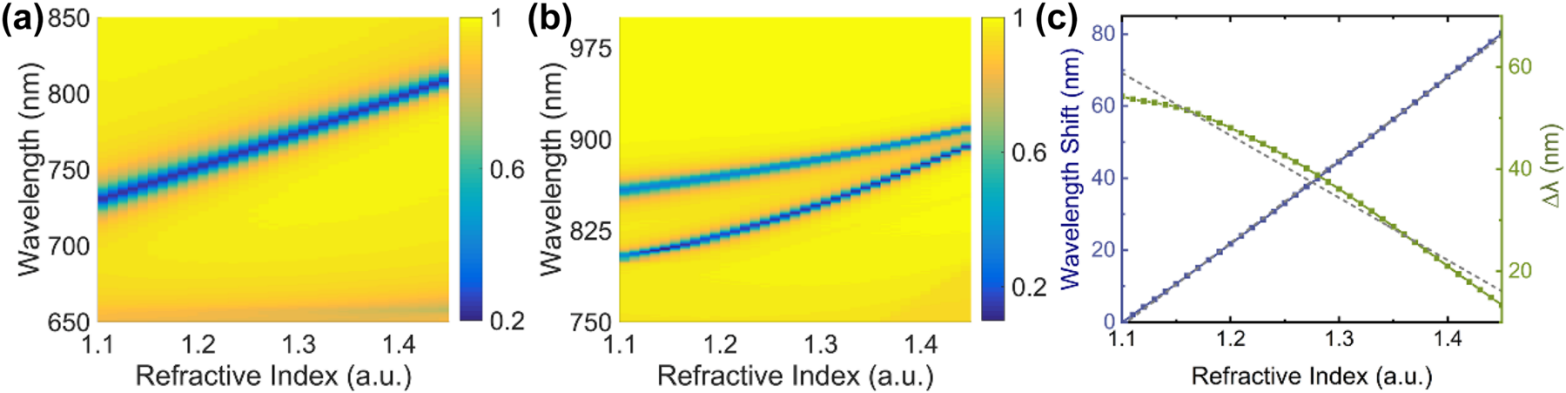
Sensing ability of two metasurfaces. (a) And (b) dependence of the transmission spectra on the environmental refractive index of the metasurfaces containing (a) two nanopillars of 100 nm height and (b) eight nanopillars of 200 nm height each period, respectively. (c) Variations of wavelength shift (blue curve) and Δ*λ* (green curve) with the refractive index for the corresponding two metasurfaces.

Figure 4 depicts the sensing ability for the designed metasurfaces. As illustrated in Figure 4(a) and (b), the transmission spectrum changes with the environmental refractive index. By evaluating the wavelength shift of the resonant peak of the control group or the spacing between the two resonances (Δ*λ*) of the target group, we can deduce the environmental refractive index. Both the wavelength shift (blue curve) and Δ*λ* (green curve) vary almost linearly with the refractive index, as shown in Figure 4(c). This near linear relationship facilitates the detection, and the fitted results are plotted with dashed lines. The corresponding slopes indicate the sensitivity of the refractive index characterization, and using this dual-resonance metasurface, the sensitivity decreases from 230 nm/RIU to 122 nm/RIU over this range. Compared to the control group, the target group shows a better performance with Q factors increased by at least 80% and 60% for the two respective resonances, for example, at an environmental refractive index of 1.3. This is also reflected by the spectral peak contrast at the resonant position, which of the target group is stronger than that of the control group. For the target group driven by dual-resonance, which contains eight 200-nm-height nanopillars each period, Q factors of the two resonances reach 415 and 364 at a refractive index of 1.45, respectively.

## 3 Experimental results and discussion

### 3.1 Metasurface fabrication

According to the geometric parameters and nanopillar arrangements, we fabricated metasurfaces in a square area with a side length of 189 μm, containing poly-Si nanopillars of 100 nm and 200 nm height, respectively. For the former case, metasurfaces containing both two and eight arranged nanopillars each period were fabricated as control groups for comparison. While for the 200-nm-height case, we only fabricated the metasurface containing eight nanopillar units in one period. The fabrication process is shown in Figure S2, Supplementary Material.

First, poly-Si was deposited on a quartz substrate by low pressure chemical vapor deposition (LPCVD) in 75 sccm silane under a gas pressure of 250 mTorr at 625 °C. We set the deposition time to 40 min and 20 min for 200- and 100-nm-thick poly-Si, respectively. A viscosifier (SURPASS 3000) was spin-coated on the poly-Si at 3000 rpm for 40 s to increase the adhesion, and an approximately 200-nm-thick electron-beam resist (ma-N 2403) was coated at 6000 rpm for 40 s and then baked at 110 °C for 60 s. Subsequently, we deposited a 5-nm-thick Al layer by thermal evaporation to increase the conductivity under a gas pressure of 4.5 × 10^−3^ mTorr and conducted the evaporation at a rate of 2 Å/s with a current of 250 A. Further, we used electron-beam lithography (EBL) to pattern the resist with a beam current of 500 pA and an exposure voltage of 100 kV. The samples were then developed in AZ300 MIF for 60 s to remove the Al layer and exposed resist. Subsequent dry etching of poly-Si was applied to the CHF_3_ and SF_6_ plasma at a ratio of 50:15 via reactive ion etching (RIE) for 140 s and 80 s, respectively. The residual resist was removed through a lift-off process in an 80 °C NMP water bath for 30 min.

**Figure 5:**
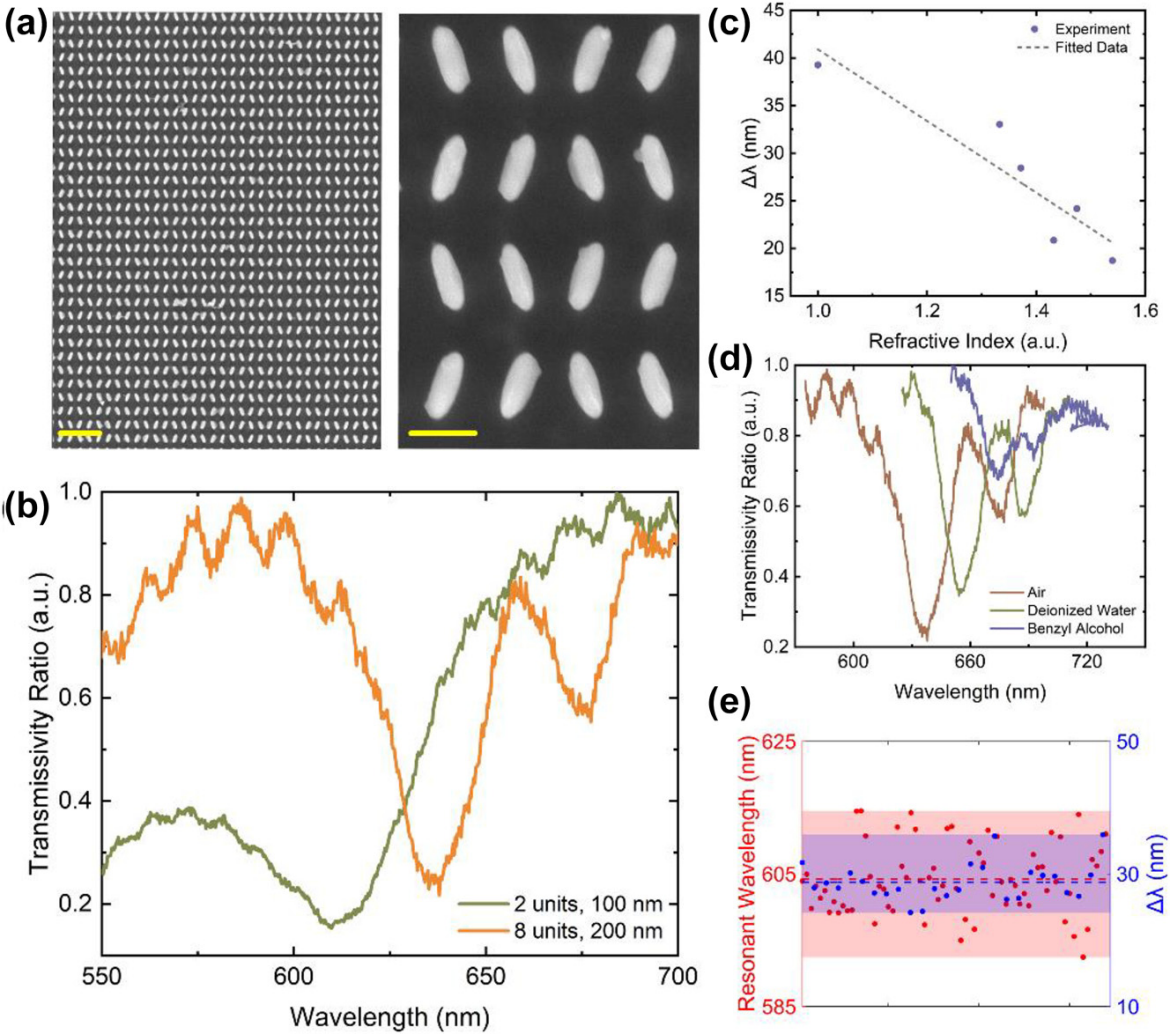
Experimental dual-resonance sensing performance of the designed metasurface. (a) SEM images of the top view of the fabricated metasurface, which contains eight 200-nm-height nanopillars in one period. The right image shows a magnified view of two periods. (b) Normalized transmission spectra of two fabricated metasurfaces, containing two 100-nm-height nanopillars and eight 200-nm-height nanopillars each period, respectively. (c) Variations of resonance peak interval Δ*λ* in six media and the fitted results. (d) Three typical transmission spectra conducted in air, deionized water and benzyl alcohol, respectively. (e) Comparison of the fluctuation ranges between the single-resonance sensing and dual-resonance sensing, which both contain eight nanopillars in one period but only differed in terms of nanopillar heights.

An additional ultraviolet lithography step was employed afterwards to create a pinhole mask. A square hole with a side length of 175 μm, slightly smaller than the processed area, was aligned to the metasurfaces, and Cr was sputtered on the unprocessed part as a mask to ensure that the spectrometer received only the light passing through the metasurfaces. The detailed fabrication process is as follows: An approximately 2.5-μm-thick resist (NR9-3000PY) was spin-coated at 4000 rpm for 40 s and baked at 120 °C for 60 s. We exposed the square area to 365-nm ultraviolet light for 18 s with an illumination intensity of 12.5 mW/cm^2^. Further, the samples were baked at 120 °C for 60 s and developed in an AZ300 MIF for 25 s. Subsequently, the processed area was coated with the photoresist, whereas the unprocessed area was unprotected. An 80-nm-thick Cr layer was deposited by magnetron sputtering as a blocking mask with favorable perpendicularity and compactness. Finally, we obtained the samples by removing the residual resist through another lift-off process in an 80 °C NMP water bath for two hours. The scanning electron microscopy (SEM) image of the metasurface shown in the left of Figure 5(a) indicates that the overall sample structures are parallel to those of the designed target group, and the SEM image on the right of Figure 5(a) depicts a partial magnification of two periods. The scale bars are 1 μm and 200 nm, respectively.

### 3.2 Experimental sensing performance

For functional performance evaluation, spectral characterizations were performed. The optical testing setup is presented in Figure S3, Supplementary Material. Figure 5(b) illustrates the corresponding normalized transmission spectra of the matasurfaces driven by single-resonance and dual-resonance in air. The green and yellow curves are in accordance with those of metasurfaces containing two 100-nm-height nanopillars and eight 200-nm-height nanopillars each period, respectively. We observed a dual-resonance spectrum when applying the target group, and the transmission dips are obviously much sharper than the control group. In experiments, the small near-period background fluctuations in the transmission spectra may be due to light interference, and they do not significantly affect the determination of the dip peaks. To verify the sensing ability of the target group, we conducted a series of experiments in six media, namely air, deionized water, ethyl acetate, 1,2-propanediol, glycerol, and benzyl alcohol, with nominal refractive indices of 1.000, 1.333, 1.372, 1.433, 1.475, and 1.540, respectively. As shown in Figure 5(c), Δ*λ* decreases almost linearly with the increase of refractive index, which is in accordance with the numerical simulations shown in Figure 4(c). Specifically, Figure 5(d) illustrates the three typical transmission spectra obtained in air, deionized water, and benzyl alcohol. Accompanied by a red shift of the resonance wavelengths, the intervals of the resonances decrease with the increase of refractive index. However, the experimental sensitivity and resonance wavelengths deviate from those of the simulations. The possible causes are discussed later.

We further performed a set of repetitive tests to demonstrate that the sensing stability could be notably improved by dual-resonance detection. To exclude the interference caused by nanopillar arrangements, the metasurface formed by eight nanopillars of 100 nm height each period is set as the control group here. We compared the transmission spectra of these two groups in air, and the tests were repeated 69 and 26 times for the control group and the target group, respectively. Figure 5(e) illustrates the stability of the two sensors. The average resonance wavelength and the average interval between the two resonance peaks are denoted by the red and blue dashed lines, respectively, and the shaded areas are the distribution regions of the data points. From these results, the sensing stability by using dual-resonance detection is almost doubled.

The deviations between the experimental results and the simulations can be attributed to many factors, including, but not limited to, the fluctuation range of the test and the difference between the reagents’ actual and nominal refractive indices. In addition to errors caused by curve fitting and fabrication accuracy, illumination conditions and spectrometer resolution are potential factors. The final deviation is an integrated result of these various above-mentioned factors.

**Figure 6:**
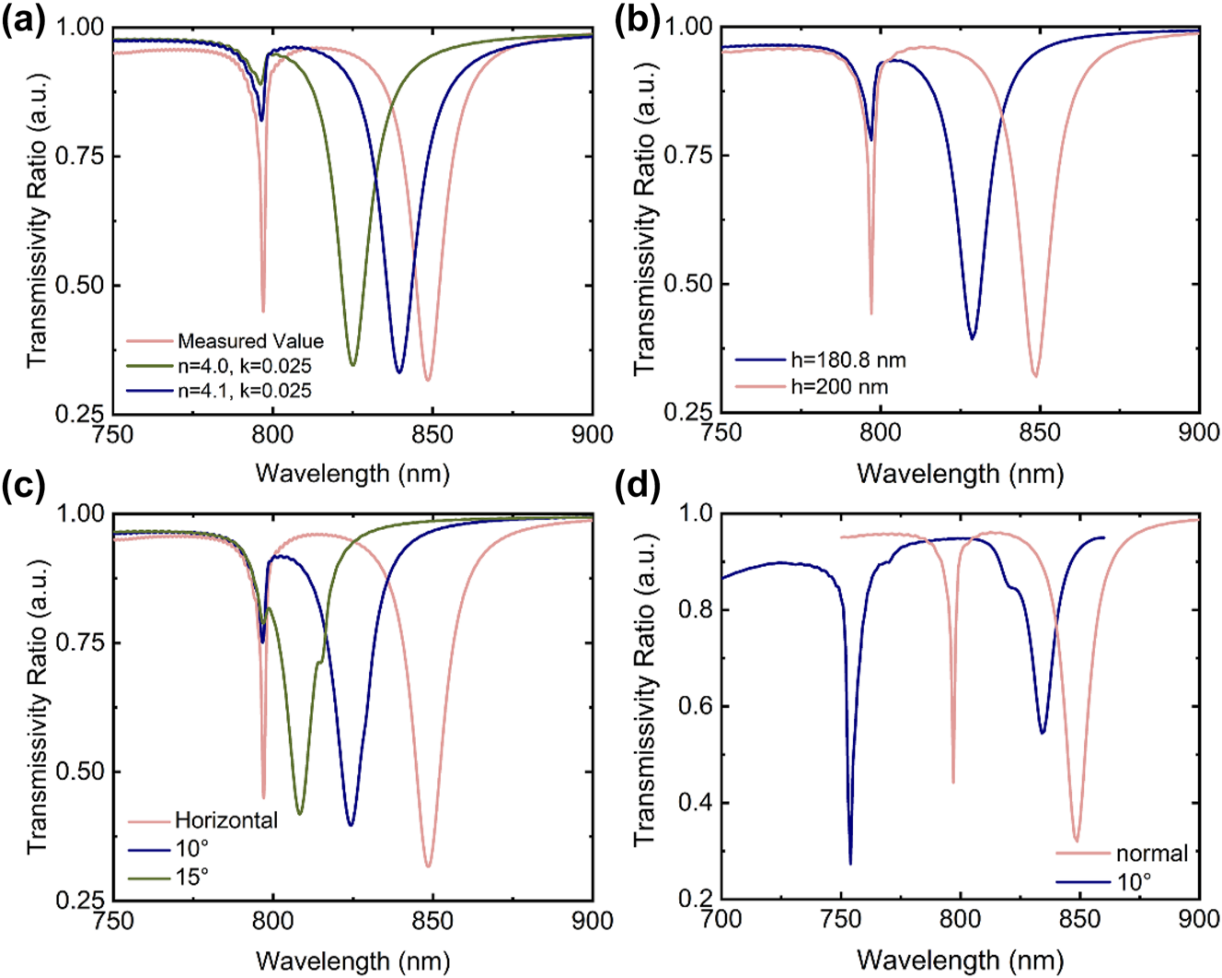
Analysis of the factors influencing the experimental and theoretical deviations. (a) Influence of material properties on the transmission spectra. (b) Transmission spectra of the metasurface with nanopillars of 180.8 nm and 200 nm height, respectively. (c) Transmission spectra with a horizontal nanopillar upper-surface and with inclined upper-surfaces of 10° and 15°, respectively. (d) Transmission spectra at normal incidence and at incidence with a pitch angle and an azimuth angle of 10°, respectively. All the metasurfaces calculated contain eight nanopillars in one period.

Although we adopted the refractive index of poly-Si with the measured value in the design, the deposition cannot be ideally uniform and the measured value may not precisely represent the property of the whole metasurface. To clarify whether the refractive index deviation has an obvious effect, we simplified the simulation here and set the refractive index (*n*) and the extinction coefficient (*k*) of poly-Si at constant values over the test waveband. The transmission spectrum shows a significant shift when *n* changes slightly. Specifically, if *n* and *k* are respectively set as 4.1 and 0.025, there will be an around 10-nm blue shift of the second resonance. This blue shift will be enlarged to around 25 nm if *n* is set as 4.0, as illustrated in Figure 6(a).

Next, we focus on the fabrication errors and illumination conditions. Each period will operate light independently when the metasurface is illuminated, and the overall sensitivity is averaged. Assume that the eight nanopillars in one period share a uniform structure and that the fabrication error is normally distributed along the major and the minor axes with a maximum fabrication error accounted for 10% of the designed parameter, a less than 2% deviation ratio of the detection sensitivity will be observed generally if the metasurface reaches a millimeter-scale. In above FDTD simulations, we only consider the dimensional deviations in the short axis and the long axis, and the influence of these fabrication errors is therefore negligible. The detailed calculations are shown in Figure S4(a) and (b), Supplementary Material. After that, we measured the height of the fabricated metasurface using an ellipsometer (Spora-GES5E), and the deposited poly-Si was found to be around 180.8-nm-thick. The simulated transmission spectra of the metasurfaces with nanopillars of 180.8 nm (navy curve) and 200 nm (pink curve) height are shown in Figure 6(b). The blue shift of the transmission spectrum is in accordance with the experimental results. Except for the fabrication errors in size, there also exist errors caused by the surface inclination. The upper surface of nanopillars is not perfectly horizontal since the nanopillars cannot be ideally etched vertically via RIE. Suppose the upper surface of the nanopillars has a slope of 10°, the resonance wavelength exhibits a blue shift of around 25 nm. Figure 6(c) indicates that this discrepancy will be enlarged to around 42 nm with a slope of 15°.

Light sources may also have a significant effect. In the FDTD simulations, we applied a flawlessly polarized plane wave, whereas, in the experiments, we illuminated the sample using an incandescent light, a complex light source. Different components of the light source are likely to exhibit different responses in the transmission spectra. Moreover, when the incident angle is adjusted, the illumination remains invariant except for the intensity; however, the transmission spectrum will change significantly. Besides, the transmission and resonance properties depend on the polarization state of the incident light because the nanopillars are not symmetric. When the incident light is polarized along the *X*-axis, strong resonance peaks are observed, whereas they are inhibited in the orthogonal polarizing direction. To verify our conjecture, we performed FDTD simulations where the metasurface was illuminated by a plane wave at incidence with both a pitch angle and an azimuth angle of 10°. Compared with the transmission spectrum at normal incidence, blue shifts of around 45 nm and 20 nm for the two resonances can be observed in Figure 6(d), which also contributes to the experimental deviations. Although the experimental angle is smaller than the set angle of 10° in the simulation, this could be an influencing factor. In addition, the larger the incident angle, the larger the deviation. This type of deviation can be eliminated using an integrated system and a more precise illumination source. However, the resonance wavelengths keep unchanged when the polarization direction of the incident light is less than 30° off the *X*-axis, which is totally controllable and can be referred to Figure S4(c) and (d) in the Supplementary Material, respectively. At last, the intrinsic spectral resolution of the spectrometer results in a broadening spectral line, and the experimental spectrum is actually the convolution of the expected spectral profile and the instrumental response function, which can reduce the Q factor.

## 4 Conclusions

In summary, we proposed a metasurface consisting of pairs of inversely tilted elliptic nanopillars. By optimizing the nanopillar height and the geometric arrangement, we obtained a dual-resonance-driven environmental refractive index sensor with a high theoretical Q factor of 415, which could be further increased with a smaller nanopillar rotation angle. Compared with conventional single-peak sensing, the spectral characterization stability doubled to a 122.20 nm/RIU sensitivity. Simultaneously, the broad detection range is very attractive, with an improvement of approximately 5000 times that of the conventional optical fiber detection [3]. The metasurface can be fabricated using mature processing techniques, and the sensitivity fluctuation is within 2%, considering the common in-plane dimensional errors in fabrication. Since the sample exhibits the same performance when illuminated from the opposite side, it could offer a more durable and stable potential as an internal structure. The detection range and working waveband can be increased by further adjusting the nanopillar size. This metasurface has promising applications in refractive index sensing, solution concentration detection, biomacromolecule identification, and cancerous cell screening.

## Supplementary Material

Supplementary Material Details
