# Supplementary material for: Dual-resonance sensing for environmental refractive index based on quasi-BIC states in all-dielectric metasurface
Source: Nanophotonics. 2023 Feb 21;12(6):1147–57. doi: 10.1515/nanoph-2022-0776 (PMC11501798; doi:10.1515/nanoph-2022-0776)
Supplement: Supplementary file 1 — Supplementary Material Details [file j_nanoph-2022-0776_suppl.docx]

Wenjie Chen,^1,2^ Ming Li,^1,2^ Wenhao Zhang,^1,2^ and Yuhang Chen^1,2,*^

^1^Department of Precision Machinery and Precision Instrumentation, University of Science and Technology of China, Hefei 230027, China

^2^Key Laboratory of Precision Scientific Instrumentation of Anhui Higher Education Institutes, University of Science and Technology of China, Hefei 230027, China

[*chenyh@ustc.edu.cn](mailto:*chenyh@ustc.edu.cn)

Dual-resonance sensing for environmental refractive index based on quasi-BIC states in all-dielectric metasurface: Supplementary Material

1 Optimization process

The optimization was conducted using the metasurface consisting of two nanopillars per period. Here, we chose six key parameters (a, b, h, θ, P_1_, P_2_) for structural optimization, and initially, the height of the nanopillars h, the rotation angle θ of each nanopillar, the major axis a and the minor axis b, and P_1_ and P_2_ were set as 100 nm, 17.5°, 280 nm, 100 nm, 500 nm and 410 nm, respectively. The optimization process was divided into four steps and from the second step onwards, the new parameters optimized from the former step replaced their initial values. To contain four or eight nanopillars arranged accordingly, as illustrated in Fig. 3(b) and (c), the optimized periods along the X- and Y-axes P_1_ and P_2_ were finally doubled, namely P_x_ = 2P_1_ and P_y_ = 2P_2_.

First, the nanopillar height h was scanned from 75 to 250 nm. Fig. S1(a) depicts that the single resonance split as h increases. Second, the rotation angle θ was scanned from 5° to 30°, while maintaining the optimal h at 200 nm. With increased θ, the contrast ratio of the resonance peaks decreases, namely, a decreased Q factor along with a blue shift, as shown in Fig. S1(b). The third step was to scan a from 140 nm to 400 nm and b from 50 nm to 240 nm. A red shift of the resonance peak is observed from Fig. S1(c) and (d), as well as an increased contrast ratio with the augmentation of each axis. Finally, the optimization of the periods along the X- and Y-axes (P_1_ and P_2_) was carried out, and both were scanned from 300 nm to 550 nm. As P_1_ increases, the spacing between the two resonances decreases with a red shift, whereas both the spacing and the resonances change oppositely as P_2_ increases, as illustrated in Fig. S1(e) and (f), respectively.

Four factors should be considered during the optimization. First, resonances must be located within the given detection range. The two resonance peaks should not overlap within the refractive index test band and their intervals should have a moderate difference. For better observation and resolution, strong spectral peak contrast is required. Moreover, fabrication should be considered to ensure that the sample preparation is feasible. Considering all these factors, we finally set h = 200 nm, θ = 17.5°, a = 240 nm, b = 80 nm, P_1_ = 450 nm and P_2_ = 350 nm. Accordingly, P_x_ and P_y_ of the target metasurface were 900 and 700 nm, respectively. The nanopillars were evenly distributed over one period, as illustrated in Fig. 3(b) and (c), respectively.


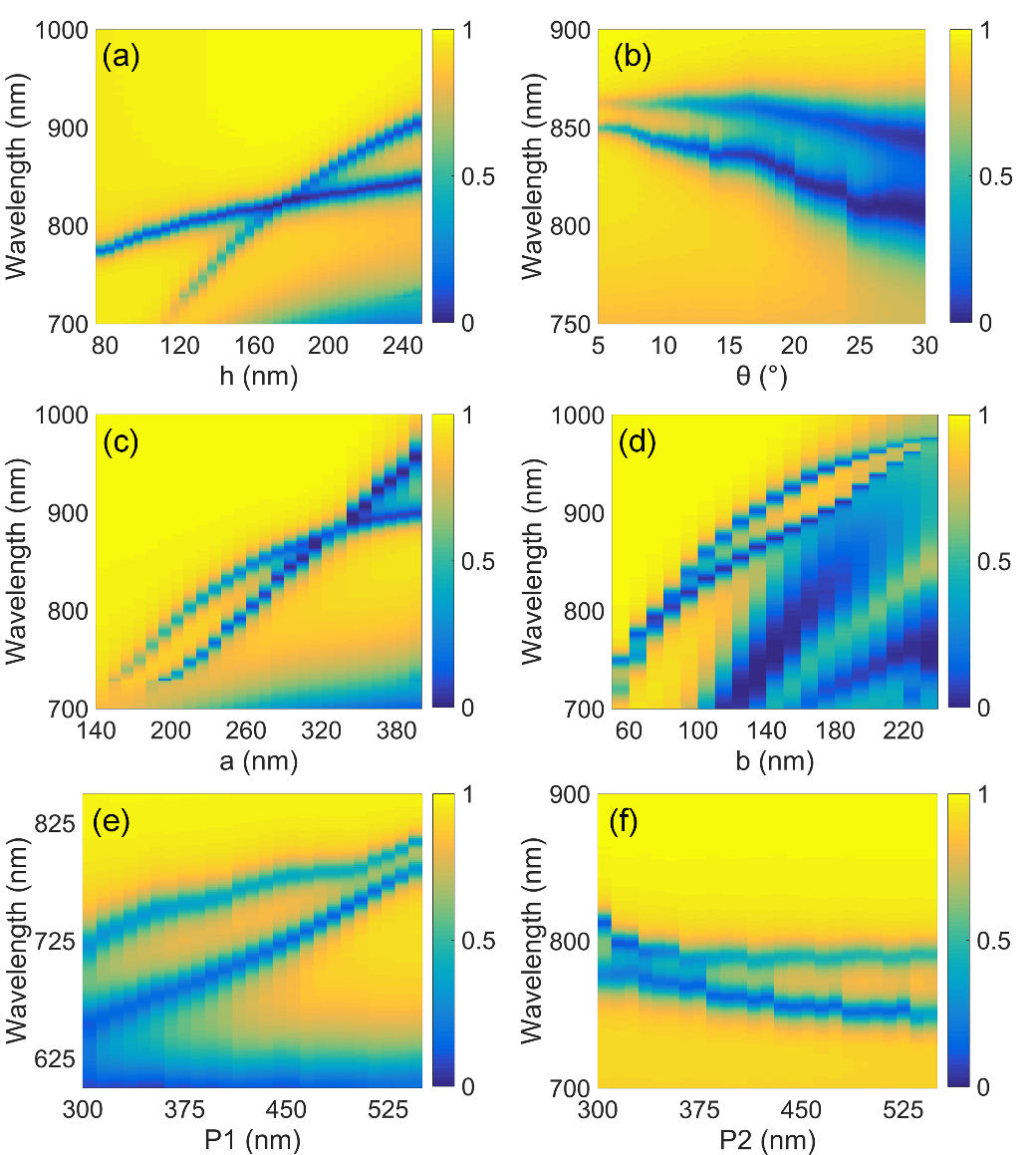


**Figure S1:** Optimization of six key parameters and the transmission spectrum maps with (a) h; (b) θ; (c) a; (d) b; (e) P_1_ and (f) P_2_.

**2 Fabrication process**

Metasurfaces containing poly-Si nanopillars of 100-nm thick and 200-nm thick were fabricated in a square area, respectively. In Fig. S2, the top row is the fabrication process of the metasurfaces, and the bottom row indicates the ultra-violet lithography procedure of a square pinhole slightly smaller to prevent the interference from the background signals.


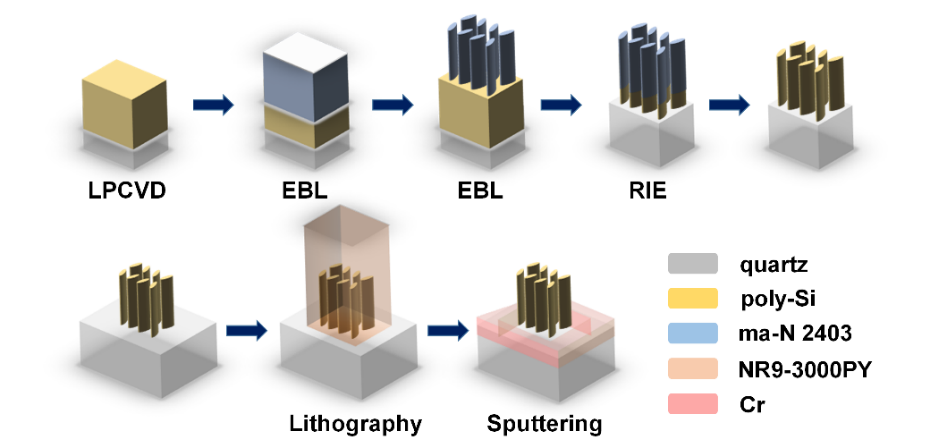


**Figure S2:** Fabrication process of the designed metasurface.

**3 Optical testing setup**

Spectral analysis is carried out using the optical testing setup sketched below to evaluate the functional performance of the metasurface. An incandescent light source illuminated the sample after a polarizer with a waveband ranging from 500 to 800 nm. The X-axis polarized light then passed through a beam splitter, where part of the light was received by the spectrometer, and part was imaged by the CCD. The metasurface was fabricated in a square area with a side length of 189 μm, above which a Cr mask with a square pinhole slightly smaller, 175 μm specifically, was sputtered to block the unwanted stray light.


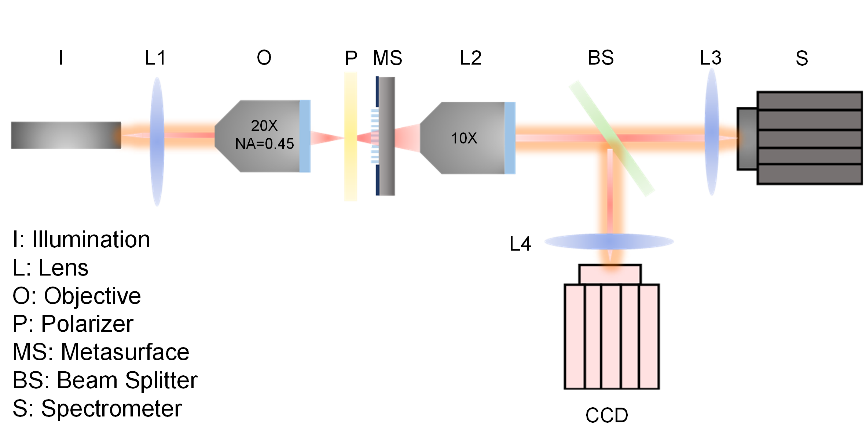


**Figure S3:** Optical testing setup

**4 Deviation caused by potential factors**

Here, we present the influence on the transmission spectra in detail of the fabrication errors along the major and minor axes and the polarization direction.

To evaluate the influence on the detection sensitivity caused by fabrication errors along the major and the minor axes, we assumed that the eight nanopillars in one period shared a uniform geometric structure, and that the fabrication error was normally distributed. The maximum fabrication error was approximated to account for 10% of the designed parameter; that is, the 3σ values were 24 and 8 nm for the major and minor axes, respectively. In the region where the two lengths may be distributed, we uniformly selected 25 points, and each point represented a possible geometric form of an elliptic nanopillar. For metasurfaces formed by these 25 nanopillars, we fitted the respective simulated transmission resonance shift versus the environmental refractive index and calculated the corresponding sensitivities. Subsequently, we performed cubic spline interpolation to obtain 8001×8001 points for better accuracy. When the metasurface is illuminated, each period will operate light independently, and the overall sensitivity will be averaged. Generally, a deviation ratio of less than 2% will be observed if there are 1500 periods working together, indicating that the influence of deviations in the major and minor axes is negligible as well. Here, 1500 periods are chosen for the calculations, which reach millimeter scale and make sense in practice. Fig. S4(a) and (b) depict the distributions of elliptical nanopillars and the corresponding deviation ratios of each period from the ideal, respectively.

Then, for both metasurfaces containing two nanopillars of 100 nm height and eight nanopillars of 200 nm height, we performed FDTD simulations with linearly polarized light of different polarization directions, that is, 0°, 10°, 15° and 30° off the X-axis, respectively. The resonance wavelengths keep unchanged in both cases, except for a slight change in the regulation depth of the transmission spectra, as shown in Fig. S4(c) and (d), respectively. The polarization direction can be totally controlled within 30° off the X-axis in the experiment, and thus the deviation caused by polarization directions can be ignorable.


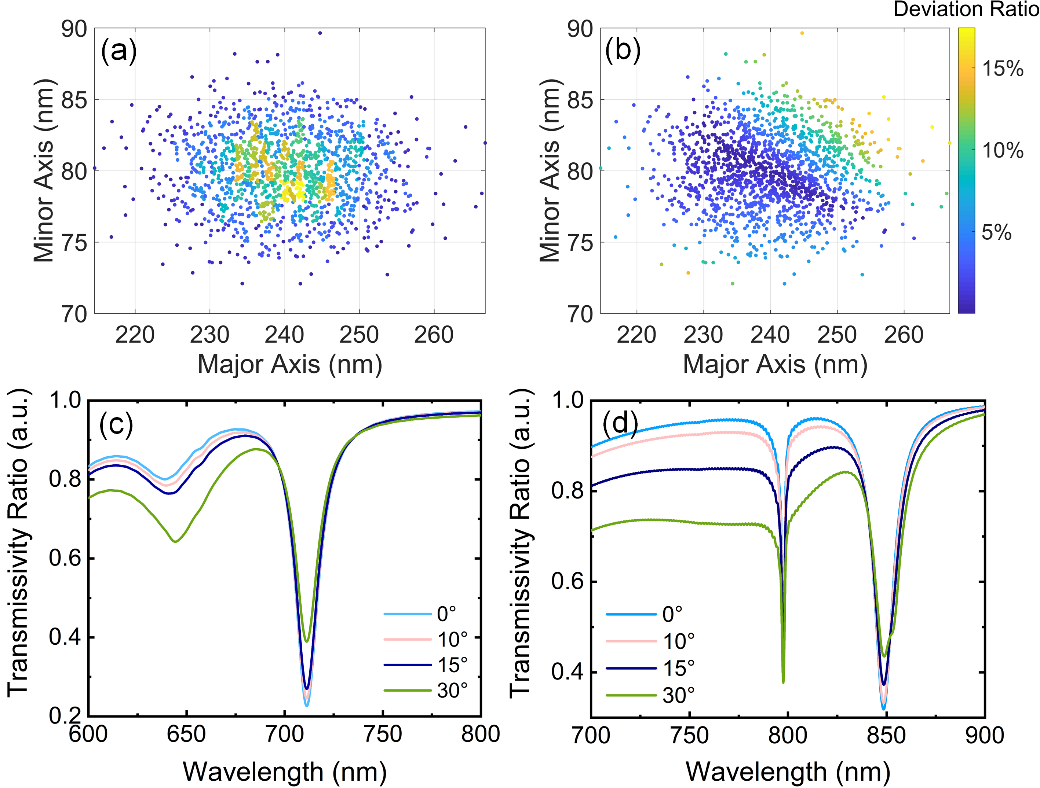


**Figure S4:** Deviation caused by potential factors. (a) Geometric shapes of the elliptic nanopillars in 1500 normally distributed periods. (b) Corresponding deviation ratios from the design. Transmission spectra of metasurfaces containing (c) two 100-nm-height and (d) eight 200-nm-height nanopillars each period with incident light polarized 0°, 10°, 15° and 30° off the X-axis, respectively.
